# Supplementary material for: Carbon Monoxide Alleviates Salt-Induced Oxidative Damage in Sorghum bicolor by Inducing the Expression of Proline Biosynthesis and Antioxidant Genes
Source: Plants (Basel). 2024 Mar 10;13(6):782. doi: 10.3390/plants13060782 (PMC10974450; doi:10.3390/plants13060782)
Supplement: Supplementary file 1 [file plants-13-00782-s001.zip › plants-2828556-supplementary.pdf]

Supplementary Materials: The following supporting information can be downloaded at: www.mdpi.com/xxx/s1, Figure S1: Effect of CO on the germination index and root length of sorghum under 200 mM salt stress.; Figure S2: Effect of CO on oxidative damage to biomolecules in sorghum under 200 mM salt stress; Table S1: Element distribution of sorghum seedlings treated with CO under salt stress; Figure S3: Effect of CO protein content in sorghum under 250 mM salt stress.

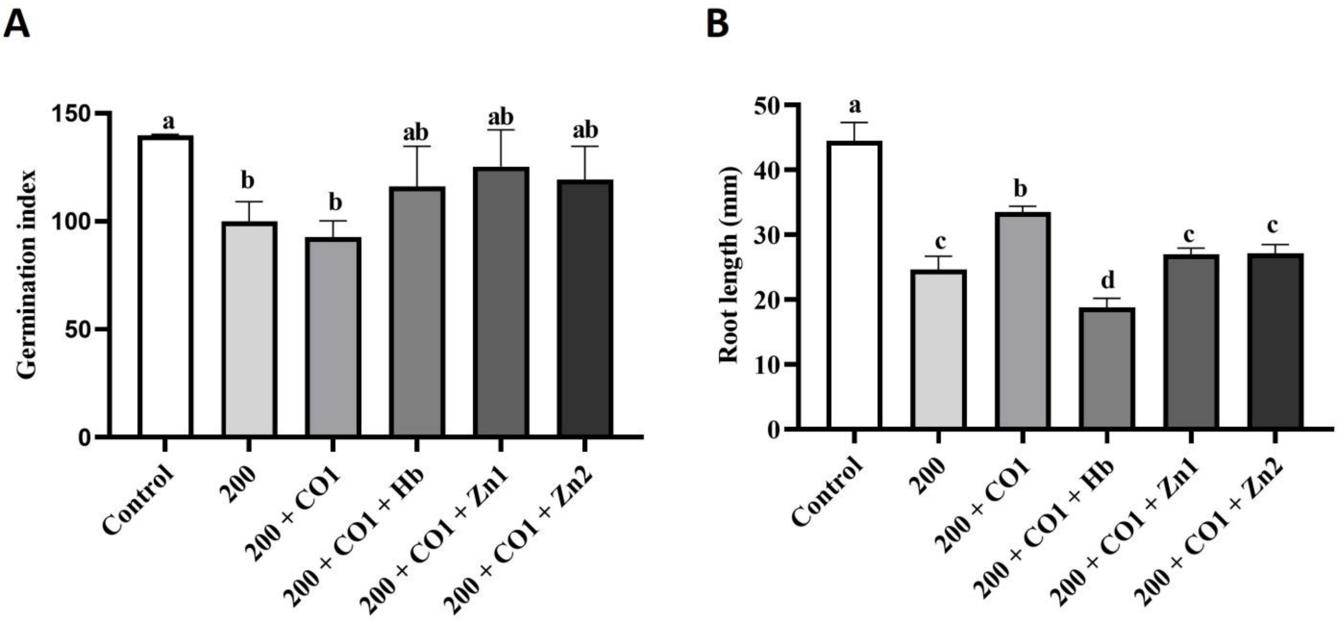

Figure S1: Effect of CO on the germination index and root length of *sorghum* under 200 mM salt stress.

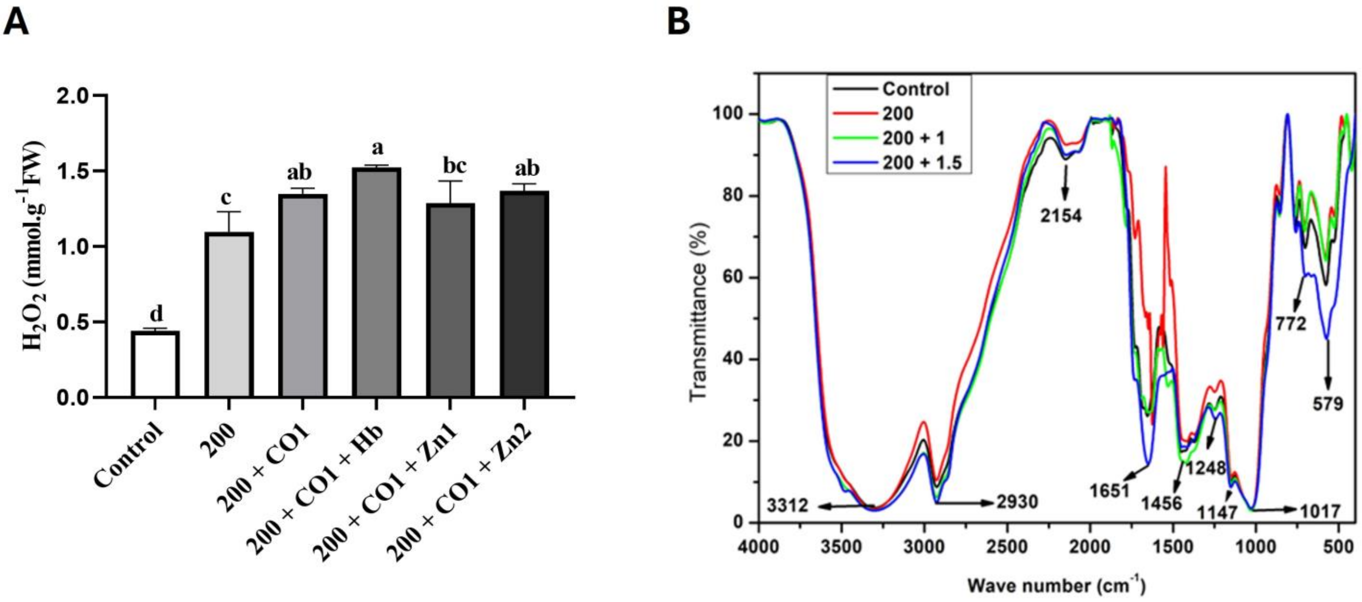

Figure S2. Effect of CO on oxidative damage to biomolecules in sorghum under 200 mM salt stress.

Table S1: Element distribution of sorghum seedlings treated with CO under salt stress.

Elements	0 mM NaCl (wt %)	200 mM NaCl (wt %)	200 + 1 Ht (wt %)	250 mM NaCl (wt %)	250 + 1 Ht (wt%)	250 + 1.5 Ht (wt%)

Na^+	0.17	**1.92**	**1.74**	**3.2**	**2.8**	**2.65**
K^+	0.6	**2.24**	**2.04**	**1.5**	**1.9**	**2.09**
			**Element Ratio**			
Na^+/K^+	**0.28**	**0.86**	**0.85**	**2.1**	**1.47**	**1.27**

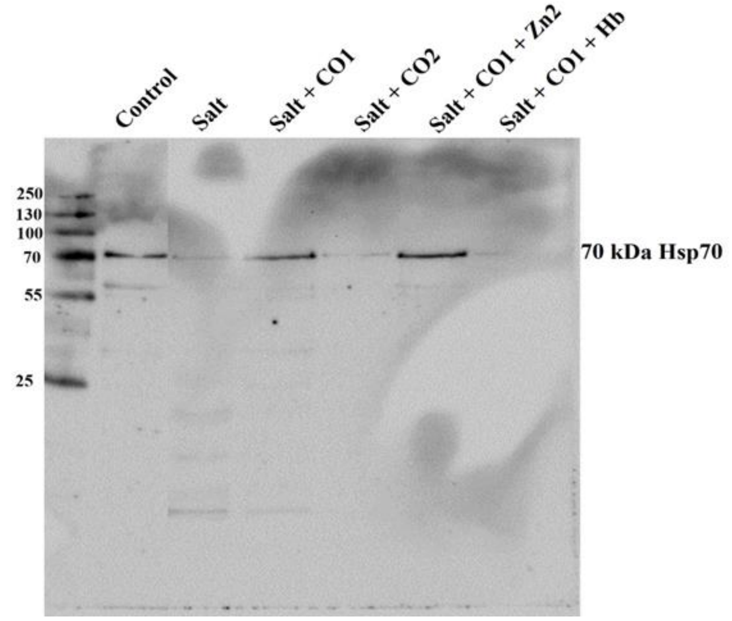

Figure S3. Effect of CO on protein content in sorghum under 250 mM salt stress. (A) Western blot analysis of Hsp70 protein; lane 1: control; lane 2: salt; lane 3: salt + CO1, lane 4: salt + CO2; lane 5: Salt + CO1 + Zn2; lane 6: Salt + CO1 + Hb; 0 mM NaCl = Control, 250 mM NaCl = Salt, Ht = CO1 (1 μM) and CO2 (1.5 μM), Hb (0.1 g/L), ZnPPIX = Zn 1 (5 μM) and Zn 2 (10 μM). Hematin (Ht), hemoglobin (Hb), and zinc protoporphyrin IX (ZnPPIX).
